# Bisphenol A Treatment Impairs Synaptic Function in Human Cholinergic Neurons

**DOI:** 10.1002/jbt.70558

**Published:** 2025-10-06

**Authors:** Anna Maria Carrese, Rossella Vitale, Manuela Turco, Francesco Aniello, Natasha Petecca, Luisa Cigliano, Emilia Vitale, Aldo Donizetti

**Affiliations:** ^1^ Department of Biology University of Naples Federico II Naples Italy; ^2^ Institute of Biochemistry and Cell Biology National Research Council (CNR) Naples Italy

**Keywords:** bisphenol A, cholinergic neurons, gene expression, SH‐SY5Y cells, synaptic activity

## Abstract

Bisphenol A (BPA) is a synthetic compound widely used in the production of polycarbonate plastics and epoxy resins, commonly found in food and beverage packaging. Exposure to BPA can occur through several routes, although the primary source is dietary. Due to its ability to cross the placental and blood−brain barriers, the adverse effects of BPA can have implications across the entire human lifespan, from embryonic development to adulthood. While its harmful effect on brain development and function has been extensively documented in animal models, its impact on human mature neurons, particularly concerning synaptic activity, remains poorly understood. In this study, we investigated the effects of BPA on a human cholinergic neuron model. BPA exposure was found to reduce neuritic complexity and alter the expression of key synaptic proteins. These structural changes were accompanied by dysregulation of activity‐regulated gene expression, indicating impaired synaptic function. Our findings suggest that even a short‐term exposure to BPA can disrupt synaptic integrity, with potential consequences for normal brain functions.

## Graphical abstract

Short‐term sublethal BPA exposure disrupts dendritic morphology, cholinergic synapse physiology, and activity‐dependent gene expression in human neurons. These alterations suggest impaired synaptic communication and plasticity, highlighting BPA's potential to affect the adult human brain even without inducing neuronal death.

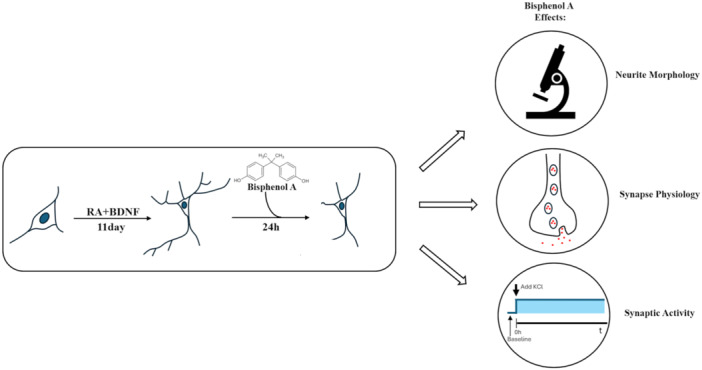

**Abbreviations**
ACHE: acetylcholinesterase
AD: Alzheimer's disease
ASD: autism spectrum disorder
BPA: bisphenol A
CHAT: choline O‐acetyltransferase
CHRNB4: cholinergic receptor nicotinic beta 4 subunit
DIV: days in vitro
E2: estradiol
GAM‐HRP: goat anti‐mouse horseradish peroxidase‐conjugated IgG
GAPDH: glyceraldehyde‐3‐phosphate dehydrogenase
GAR‐HRP: goat anti‐rabbit horseradish peroxidase‐conjugated IgG
LncRNA: long non‐coding RNA
MAR‐HRP: mouse anti‐rabbit horseradish peroxidase‐conjugated IgG.
PSD 95: postsynaptic density protein 95
RA: retinoic acid
SCZ: schizophrenia
SYP: synaptophysin
SYT: synaptotagmin
VACHT: vesicular acetylcholine transporter

## 1 Introduction

Bisphenol A (BPA) is one of the most widespread environmental endocrine disruptors [1], released from various everyday products and absorbed through multiple exposure routes [2]. Human exposure occurs mainly through the diet, not only through the ingestion of food packaged in plastics and cans, but also through seafood and freshwater fish contaminated with BPA [3]. BPA‐containing polymers can undergo hydrolysis at high temperatures and under acidic or basic conditions, resulting in the leaching of BPA into food and beverages [4]. Although it has weak estrogenic activity, BPA is considered a “mimic hormone” of estradiol (E2) [5]. Moreover, it also exerts estrogen‐independent effects [6, 7, 8], which have been linked to many adverse outcomes. The brain is a primary target of BPA, both during development and in adulthood, due to its ability to cross the blood−placental and blood−brain barriers, thanks to its lipophilic nature [9, 10]. Neural development is particularly vulnerable to endocrine disruptors. Prenatal BPA exposure has been shown to cause abnormalities in the fetal hippocampus and reduce hippocampal spine synapses in nonhuman primates [11, 12]. BPA interferes with key differentiation processes, including neurite extension and branching, synaptic formation, neurotransmitter expression, and neuronal survival in rat hypothalamic neurons [13]. It is also linked to oxidative stress and neuroinflammation, contributing to neurodegeneration [14, 15, 16, 17]. BPA is considered a potential environmental factor in the onset of neurodegenerative diseases, including Alzheimer's disease (AD) [18]. In particular, it has been shown to trigger AD‐like neurotoxicity by increasing pathological protein levels, a process associated with the impaired insulin‐IR‐IRS1 signaling [19]. Although several studies have shown that postnatal BPA exposure impairs synaptic plasticity in the prefrontal cortex and hippocampus of nonhuman primates [20, 21], rats [22, 23], and mice [24], these findings are primarily derived from animal models. BPA neurotoxicity has been extensively investigated through both in vivo studies in animals and in vitro approaches using cultured neurons of human and nonhuman origin. However, despite their value in revealing general effects, nonhuman models are limited by species‐specific differences that challenge the direct extrapolation of these results to humans. This is especially critical in the context of brain‐related disorders, where interspecies variability is particularly pronounced [25]. Therefore, the use of human‐derived models becomes essential for gaining more accurate insights into the neurotoxic potential of BPA. In this context, the SH‐SY5Y human neuroblastoma cell line has proven to be a suitable in vitro model to explore the molecular mechanisms underlying neuronal responses to various environmental pollutants [26, 27]. Nonetheless, most studies have focused on undifferentiated SH‐SY5Y cells, which do not fully recapitulate mature neuronal features. To overcome this limitation, we employed a differentiated cholinergic‐like model of SH‐SY5Y cells to investigate the subtle effects of short‐term, sublethal BPA exposure. Our findings reveal that BPA disrupts dendritic branching, a process essential for synaptic connectivity, and modulates the expression of plasticity‐related genes, suggesting that even sublethal exposure could impact synaptic function in the adult human brain without inducing neurotoxicity.

## 2 Materials and Methods

### 2.1 Cell Culture

SH‐SY5Y (human neuroblastoma, ECACC, Porton Down, SP4 0JG Salisbury, UK) cell line was grown and propagated in Dulbecco's Modified Eagle's Medium (DMEM, EuroClone, Milan, Italy) combined with Ham's F12 (EuroClone, Milan, Italy), supplemented with 2 mM l‐glutamine (EuroClone, Milan, Italy), a solution of 1% penicillin/streptomycin (EuroClone, Milan, Italy), and 15% fetal bovine serum (FBS, EuroClone, Milan, Italy). Cells were cultured and maintained in a 5% CO_2_ humidified incubator at 37°C.

### 2.2 Differentiation and BPA Treatment of Cell Cultures

SH‐SY5Y cells were differentiated by incubation in a low serum (1.5%) medium containing 10 µM retinoic acid (ATRA) (RA, SIGMA‐Aldrich, St. Louis, MO, USA) and 50 ng/mL brain‐derived neurotrophic factor (BDNF) (PeproTech, London, UK). 4 × 10^5^ and 1 × 10^6^ cells were seeded in 35 and 60 mm plates, respectively, and stimulated by the differentiation medium, which was refreshed every 2 days. Cells have been differentiated for 12 days. The differentiation process was monitored by the LEICA DMi8 microscope. BPA (99% purity) was obtained from Sigma‐Aldrich (St Louis, Missouri, USA). A 100 mM stock solution of BPA was prepared by dissolving BPA in ethanol 100%, and stored at −20°C. The stock solution was then diluted in water to obtain an 80 mM working solution. On Day 11 of differentiation, the culture medium was replaced with fresh medium containing BPA at a final concentration of 80 μM, or 0.08% ethanol as a vehicle control.

### 2.3 Morphometric Analysis

Image processing and analysis were performed using Fiji software (ImageJ). The NeuronJ plug‐in was employed to quantify the number and length of neurites [28] from phase‐contrast images of control and BPA‐treated cells, acquired at ×20 magnification using a LEICA DMi8 microscope. Primary and secondary neurites were plotted semiautomatically using three images per experimental condition in each of three independent biological replicates (nine images in total), then the number and total length of neurites were normalized to the number of neurons to obtain the number of neurites per neuron in each image. Finally, to analyze the branching of neurites, the nodes (the points of the primary neurites from which the secondary neurites branch) and the total number of cells were counted for each image by using a preformed grid, obtaining the number of nodes per cell.

### 2.4 Depolarization Protocol

At 12 days, cells were stimulated to induce depolarization by adding KCl depolarization solution (170 mM KCl, 10 mM Hepes pH 7.4, 1 mM MgCl_2_, 2 mM CaCl_2_) directly into the neuronal culture medium to a final concentration of 31%, corresponding to a final KCl concentration of 55 mM. Cells were then incubated for 8 h as previously described [29]. In particular, control and BPA‐treated cells were collected at time 0 and after 8 h of depolarization for total RNA extraction.

### 2.5 RNA Isolation, Retrotranscription, and Quantitative PCR Analysis

Total cellular RNA isolation, retrotranscription, and qPCR were performed as reported by Carrese et al [29]. Briefly, RNA was isolated using Ribospin (GeneAll Biotechnology Co. Ltd., Korea), and 1 μg of RNA was reverse‐transcribed into cDNA using Luna Script RT SuperMix (New England Biolabs, Ipswich, MA, USA). qPCR was performed on three independent biological replicates, in technical duplicate for each biological replicate using the SYBR green (SYBR Green GDSBio, Guangpu East Road, Huangpu District, Guangzhou, Guangdong, China) method. The reaction mixture contained 20 ng of cDNA template and 400 nM of each forward and reverse primer in a final volume of 15 μL. The following primer pairs were used: *ACHE* (NM_001367919.2), forward (5’‐CTCAGCGCCACCGACAC‐3’) and reverse (5’‐CTGGTTCTTCCAGTGCACCA‐3’); *BDNF* (NM_170734.4), forward (5’‐ACACAAAAGAAGGCTGCAGG‐3’) and reverse (5’‐TGCTATCCATGGTAAGGGCC‐3’); *CHAT* (NM_001142929.2), forward (5’‐CGAGGAGAGCAGGTCCACA–3’) and reverse (5’‐TTTGCTGCCATCTTACGGGG‐3’); *CHOP* (NM_001195053.1), forward (5’‐ACCTCCTGGAAATGAAGAGGAAG‐3’) and reverse (5’‐CAGTCAGCCAAGCCAGAGAA‐3’); *CHRNB4* (NM_000750.5), forward (5’‐CGGGCGCGGGAACTG‐3’) and reverse (5’‐GCTGGGCGGATCAGGTTATT‐3’); *LINC00473* (NR_026860.1), forward (5’‐AAACGCGAACGTGAGCCCCG‐3’) and reverse (5’‐ CGCCATGCTCTGGCGCAGTT‐3’); *NR4A1* (NM_001202234.2), forward (5’‐ CACAGCTTGCTTGTCGATGT‐3’) and reverse (5’‐GGTTCTGCAGCTCCTCCAC‐3’); *VACHT* (NM_003055.3), forward (5’‐TCATCGACCGCATGAGCTAC‐3’) and reverse (5’‐GGCGAACAGGACTGTAGAGG‐3’). The PCR conditions included a denaturation step (95°C for 10 min) followed by 40 cycles of amplification and quantification (95°C for 35 s, 60°C for 1 min). The relative gene expression levels were normalized to the reference gene Glyceraldehyde‐3‐Phosphate Dehydrogenase (*GAPDH*) (NM_002046.7) with the following primers: forward (5’‐AAAATCAAGTGGGGCGATGC‐3’) and reverse (5’‐GGCAGAGATGATGACCCTTT‐3’) and calculated by the $2^{-\Delta \Delta C_{t}}$ method. The data are reported in the graph as Log_2_FC.

### 2.6 Western Blot Analysis

At the end of incubation, media samples were discarded, while cells were extensively washed with DMEM before being lysed in 70 µL of RIPA buffer (150 mM NaCl, 50 mM Tris‐HCl pH 8.0, 0.5% sodium deoxycholate, 0.5% NP‐40, 0.1% SDS pH 8.0), as previously reported [30]. The buffer was supplemented with protease (1:500, v/v) and phosphatase (1:100, v/v) inhibitor cocktails. After centrifugation (12,000*g*, 40 min, 4°C), the protein concentration in the supernatants was determined as previously reported [30]. Specifically, 30 µg of cell protein extracts were resolved by denaturing and reducing electrophoresis SDS‐PAGE [30] on either 12.5% or 10% polyacrylamide gels. Proteins blotting onto PVDF membrane (GE Healthcare; Milan, Italy), as well as washing and blocking steps were performed according to established protocols [31]. Following blocking, the membranes were incubated overnight, at 4°C with primary antibodies against SYP (AB9272; Merk‐Millipore; dilution 1:80,000), SYT (#14558; Cell Signaling Technology; dilution 1:1000), PSD‐95 (#2507; Cell Signaling Technology; dilution 1:1000), PARP‐1 (AB‐83632; Immunological Science; dilution 1:1000), and CASP‐3 (sc‐271028; Santa Cruz; dilution 1:1000). After washing, membranes were incubated for 1 h at 37°C with the appropriate peroxidase‐conjugated secondary antibodies: GAR‐HRP IgG (GtxRb‐003‐DHRPX; Immunoreagents; 1:15,000–1:60,000), MAR‐HRP IgG (7074S; Cell Signaling Technology; dilution 1:1000), GAM‐HRP IgG (98‐32022023; Immunoreagents; dilution 1:1000). For loading control, after detection of each target protein, membranes were stripped [31] and incubated overnight 4°C with mouse anti‐β‐actin (A2228; Sigma‐Aldrich; dilution 1:1000) or GAPDH (5174S; Cell Signaling Technology; dilution 1:1000) antibodies, followed by the respective HRP‐conjugated secondary antibodies: GAM‐HRP IgG (GtxMu‐003‐DHRPX; Immunoreagents; dilution 1:60,000) and MAR‐HRP IgG (7074S; Cell Signaling Technology; dilution 1:1000). Signal detection was carried out using the Excellent Chemiluminescent Kit Westar Antares (Cyanagen s.r.l., Bologna, Italy). Densitometric analysis was performed on images acquired via Chemidoc or digitalized X‐ray films using the Un‐Scan‐It gel analysis software (Silk Scientific, UT, USA).

### 2.7 Statistical Analysis

Statistical analyses were performed using GraphPad Prism software (San Diego, CA, USA). Data from independent biological replicates are expressed as mean ± SEM. For comparisons between two groups, a two‐tailed Student's *t*‐test was used. For comparisons involving more than two groups, one‐way ANOVA followed by Tukey's post hoc test was applied. A *p* < 0.05 was considered statistically significant.

## 3 Results

### 3.1 BPA Exposure Induces Stress and Alters the Neurite Morphology

**Figure 1:**
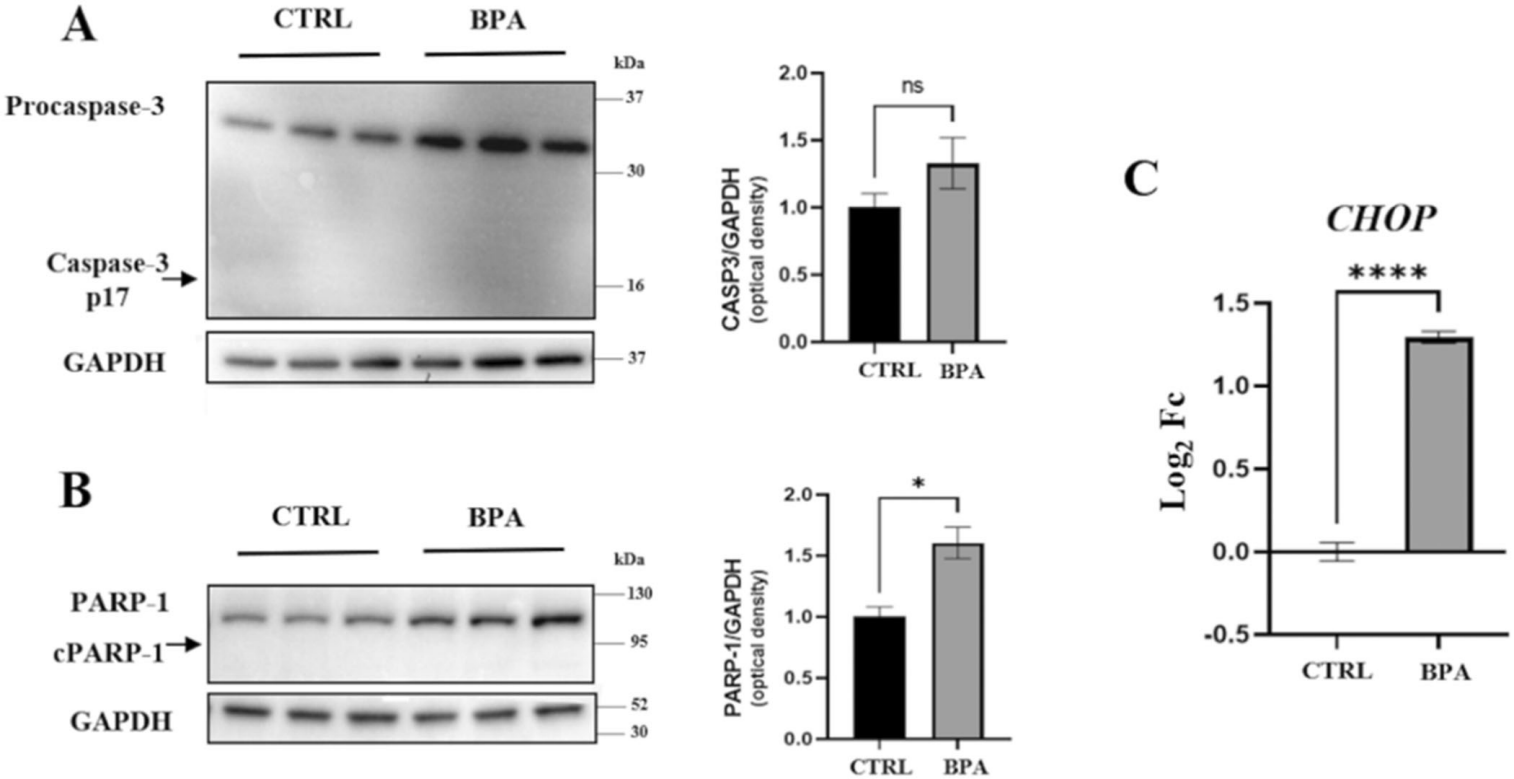
Densitometric analysis of CASP3 (A) and PARP‐1 (B) protein levels and expression levels of *CHOP* gene (C) in CTRL and BPA‐treated cells. The level of glyceraldehyde‐3‐phosphate dehydrogenase (GAPDH) protein was used as a loading control for protein normalization in western blot analysis. For both CTRL and BPA‐treated samples, each lane represents an independent biological replicate of the experiment. Gene expression level was performed on three independent biological replicates, was normalized to the reference transcript *GAPDH* and calculated as Log_2_FC. Values are reported as mean ± SEM. ns: not significant, *p ≤ 0.05, ****p ≤ 0.0001. Uncropped gels are provided in the Figure S1.

The neurotoxic effects of BPA have been widely documented in previous studies [32]. In this study, we specifically aimed to use a sublethal dose and experimental conditions that would allow us to explore cellular responses in the absence of evident neurotoxicity, to gain insight into more subtle or early molecular alterations. To identify a suitable concentration for assessing sublethal effects during neuronal differentiation, we conducted preliminary assays in which cells were exposed to 20, 80, and 100 µM BPA in the presence of RA and BDNF for the first 6 days of differentiation (data not shown). Of these concentrations, 100 µM induced evident cytotoxicity, whereas 80 µM was the highest concentration that did not cause evident cell death in our system. Therefore, 80 µM was selected for further experiments as a sublethal dose positioned just below the cytotoxic threshold. This choice was supported by the findings of Sendra et al. [33], who reported that a 24 h exposure to 80 µM BPA in 4 days‐differentiated SH‐SY5Y did not significantly affect cell viability, confirming that this concentration is sublethal under similar experimental conditions. To assess the impact of sublethal BPA exposure on mature neurons, SH‐SY5Y cells were differentiated into cholinergic neurons for 12 days, as previously reported by Carrese et al. [29]. The differentiated cells were exposed to BPA or treated with ethanol as a vehicle control for 24 h. The number of cells remained unaltered, indicating that the applied concentration and exposure time did not exert a cytotoxic effect. This finding is corroborated by the absence of CASP‐3 and PARP1 cleaved bands revealed by western blot analysis (Figure 1A,B). Differently, the analysis of full‐length PARP1 demonstrated a significant protein level increase (Figure 1B). This finding highlights that BPA treatment may induce cellular stress without the hallmarks of apoptosis under the specified experimental conditions. CHOP mRNA is a well‐established readout of cellular stress, with increased expression reported under conditions of endoplasmic reticulum stress and other stress stimuli [34, 35, 36]. The results of the analysis of *CHOP* mRNA levels confirm the existence of a state of cellular stress, since a significant increase was observed in the levels of this transcript following treatment with BPA (Figure 1C).

**Figure 2:**
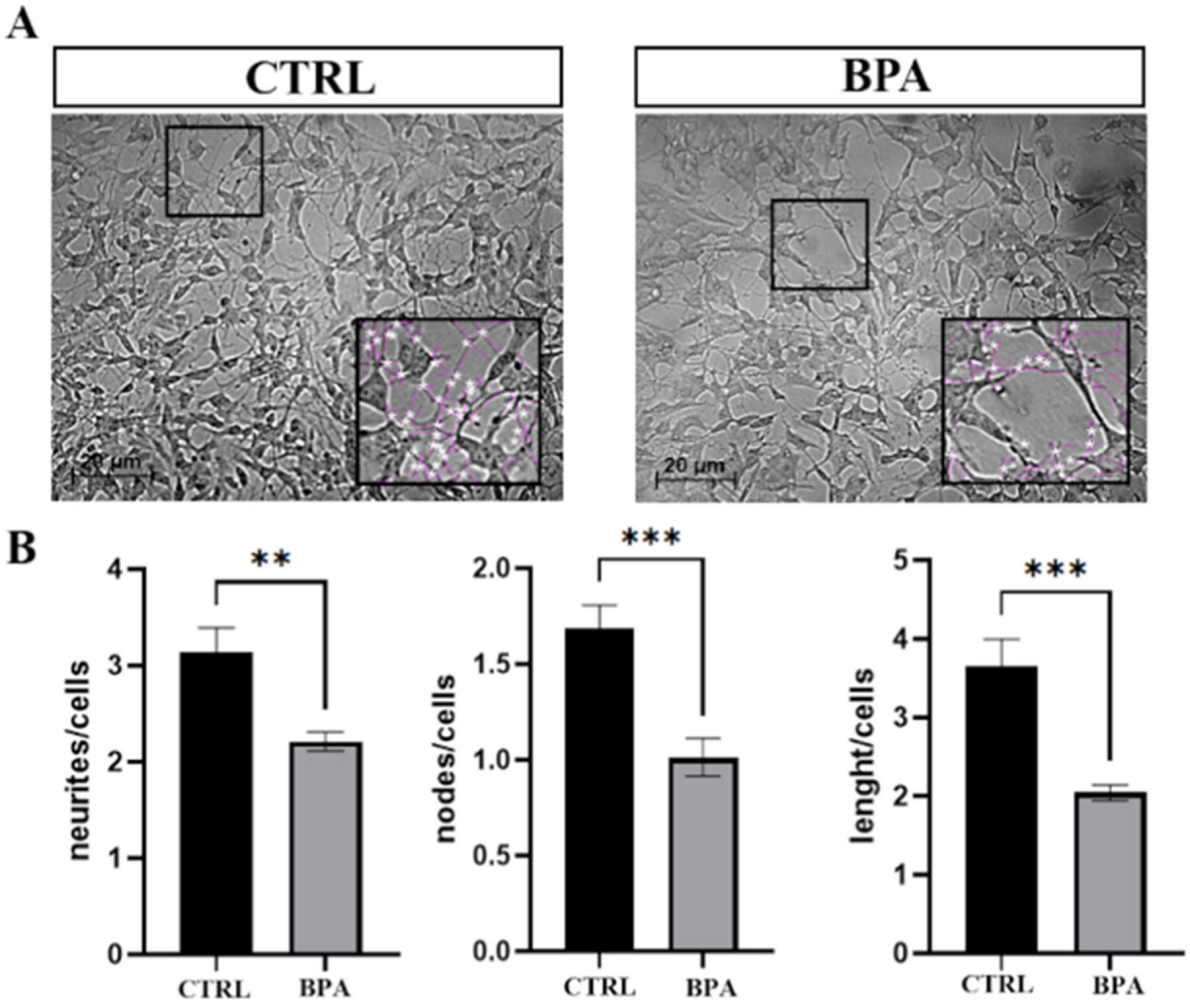
Morphological comparison between phase‐contrast images of neuron‐like cells at 12 days of differentiation treated with EtOH (CTRL) or BPA (A); graphs of morphometric parameters of CTRL and BPA‐treated cells (B). Analyses were performed on three independent biological replicates (nine images in total). Values are reported as mean ± SEM. **p ≤ 0.01, ***p ≤ 0.001.

The morphometric analysis revealed an impact on neurite morphology, as demonstrated by the magnification of bright field images (Figure 2A). All three morphometric parameters (neurites number and length, and nodes number) exhibited a significant reduction in values in BPA‐treated neurons compared to the control (Figure 2B).

### 3.2 BPA Exposure Alters Synaptic Protein Expression

**Figure 3:**
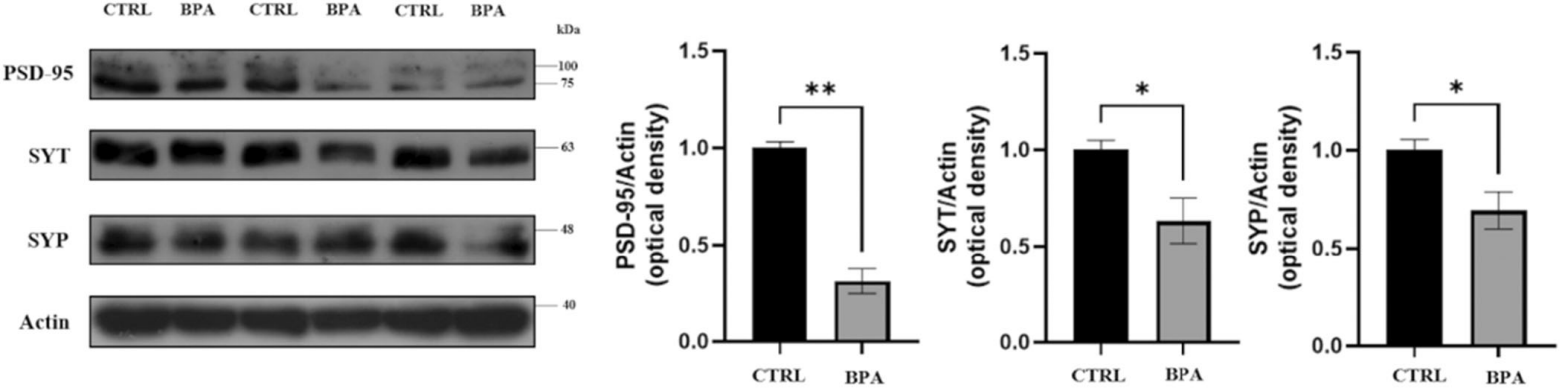
Densitometric analysis of PSD95, SYT, and SYP protein levels of CTRL and BPA‐treated cells. The level of ACTIN protein was used as a loading control for protein normalization in western blot analysis. For both CTRL and BPA‐treated samples, each lane represents an independent biological replicate of the experiment. Values are reported as mean ± SEM. *p ≤ 0.05, **p ≤ 0.01. Uncropped gels are provided in the Figure S1.

To assess the potential impact on synaptic activity, we conducted a detailed analysis of the protein levels of two presynaptic proteins, namely synaptotagmin (SYT) and synaptophysin (SYP), and the postsynaptic density protein 95 (PSD‐95), which have been identified as playing a pivotal role in synaptic plasticity [37]. The level of all the analyzed markers was found to be significantly reduced in BPA‐treated cells (Figure 3), thereby providing support for an alteration in the number and/or structure of synapses.

### 3.3 BPA Effect on Acetylcholine (ACh) Homeostasis

**Figure 4:**
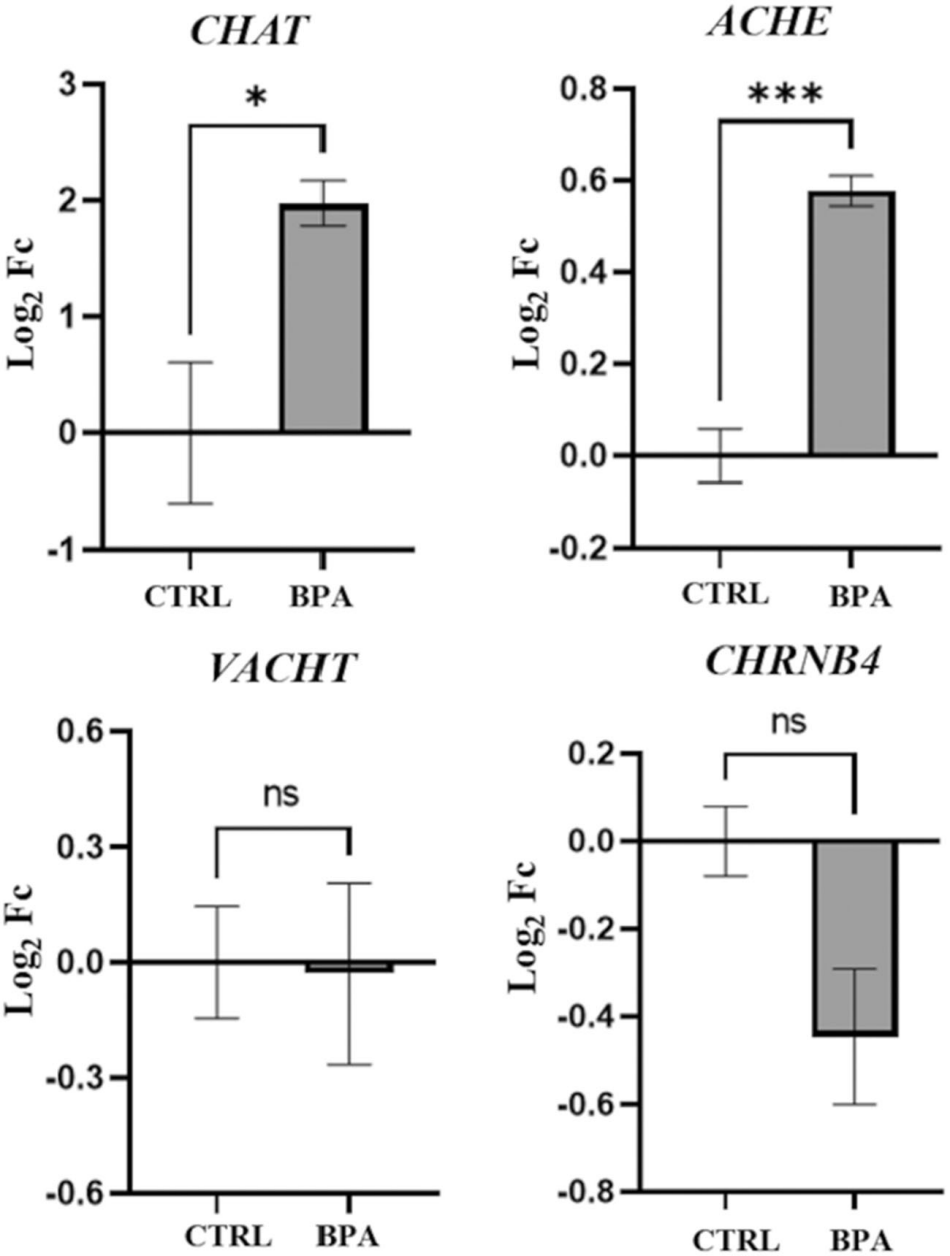
Expression levels of genes *CHAT*, *ACHE, VACHT*, and *CHRNB4* in CTRL and BPA‐treated differentiated SH‐SY5Y cells. Gene expression level was performed on three independent biological replicates, normalized to the reference transcript *GAPDH,* and calculated as Log_2_FC. Values are reported as mean ± SEM. ns: not significant; *p ≤ 0.05; ***p ≤ 0.001.

To investigate whether BPA treatment affects ACh metabolism, we analyzed the expression of key genes involved in its synthesis, degradation, storage, and signaling. ACh levels are tightly regulated by multiple processes, including their synthesis, storage in vesicles, and enzymatic degradation. In BPA‐treated neurons, transcript levels of the synthesizing enzyme choline O‐acetyltransferase (CHAT) and the degrading enzyme acetylcholinesterase (ACHE) were significantly altered compared to control cells, suggesting a disruption in the normal turnover of ACh. In contrast, no changes were observed in the expression of the vesicular acetylcholine transporter (VACHT) or of nicotinic ACh receptors, specifically the beta subunit (CHRNB4) (Figure 4).

### 3.4 Effect of BPA on Synaptic Activity

**Figure 5:**
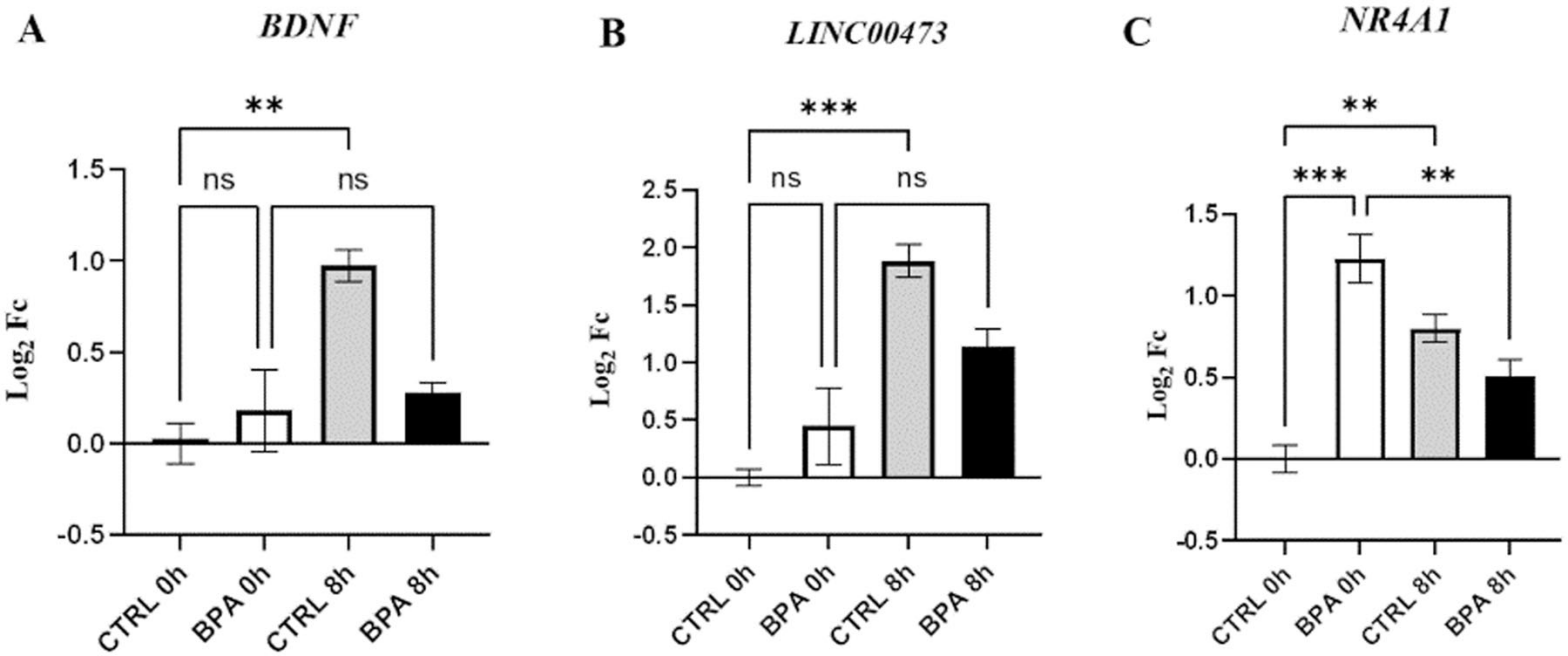
Expression levels of *BDNF* (A), *LINC00473* (B), and *NR4A1* (C) genes in CTRL and BPA‐treated differentiated SH‐SY5Y cells upon KCl stimulation. Gene expression level was performed on three independent biological replicates, normalized to the reference transcript *GAPDH,* and calculated as Log_2_FC. Values are reported as mean ± SEM. ns: not significant; **p ≤ 0.01; ***p ≤ 0.001.

The previous data collectively indicate that BPA may impact on the structure and function of synapses. To assess whether synaptic activity is dysregulated by BPA, we employed a depolarization model based on treatment with high concentrations of KCl to simulate synaptic activity. Following 24 h of BPA treatment, neurons were subjected to depolarization with KCl. Based on our previously published data on genes that respond to KCl stimulation [29], we analyzed the expression of the neurotrophin *BDNF*, the transcription factor *NR4A1*, and the primate‐specific long non‐coding RNA *LINC00473*. The expression level analysis was conducted after 8 h of KCl stimulation. As expected, the expression levels of all analyzed transcripts increased upon KCl treatment, as shown in Figure 5A−C. In contrast, the increase in *BDNF* and *LINC00473* transcripts was suppressed in BPA‐treated neurons (Figure 5A,B). Conversely, *NR4A1* exhibited a higher transcript level following BPA treatment before depolarization compared to the control. Following depolarization, the transcript level decreased in neurons treated with BPA (Figure 5C).

## 4 Discussion

The exposure of humans to BPA has increased significantly in recent years due to the extensive incorporation of this pollutant into everyday commodities [38, 39]. Given its widespread presence and capacity to cross the blood−brain barrier, there are growing concerns about the potential neurotoxic effects of BPA, particularly during critical windows of brain development. In fact, emerging evidence suggests that acute or chronic exposure to BPA causes detrimental effects on cognition and memory, affecting synaptic plasticity, in experimental animals exposed during the postnatal period [18]. In the present work, we wanted to investigate the effect of BPA on human cholinergic neurons, which are particularly relevant in various brain disorders and neurodegenerative diseases including AD. The model is based on differentiated SH‐SY5Y under RA and BDNF stimulation for 12 days to obtain mature cholinergic neurons. This experimental setup was specifically designed to investigate the impact of sublethal BPA exposure on functionally mature neurons, mimicking conditions relevant to adult exposure scenarios. In particular, we used a nontoxic dose and time exposure to reveal the effects of BPA on neuronal function. Exposure to 80 μM BPA for 24 h did not induce apoptosis, as shown by microscopy and absence of cleaved CASP3 and PARP1, in agreement with previous data on similarly differentiated SH‐SY5Y cells [33]. The western blot analysis of PARP1 revealed an increase in the full‐length protein level, indicating a cellular response to stress conditions. This was also confirmed by the increase in *CHOP* mRNA levels in BPA‐treated neurons. We next examined whether BPA exposure could affect key components of cholinergic synaptic function.

Although previous studies in murine models reported reductions in cholinergic transmission and cognitive deficits following chronic BPA exposure [40, 41], our data show an increase in the transcript levels of *ACHE* and *CHAT* after 24 h of exposure. This discrepancy could be attributed to multiple factors, including differences in cell type (human vs. murine), stage of differentiation, or the toxicological response at specific concentrations. It is noteworthy that the concentrations used in our study, although comparable to those employed by Moyano et al. [41], induce significant cell death in SN56 cells, which may reflect species‐ or model‐specific susceptibility. Despite the differences, both our findings and those of previous studies converge on a common point: BPA perturbs the cholinergic system, supporting the notion that it is a sensitive and vulnerable target of environmental neurotoxins.

Interestingly, in our model, no significant alterations were observed in the expression of *VACHT* or *CHRNB4*, indicating that vesicular loading and receptor density may require longer or repeated exposures to exhibit transcriptional changes, or may be regulated post‐transcriptionally. The early upregulation of *CHAT* and *ACHE* transcripts might reflect a compensatory mechanism in response to an initial disturbance in cholinergic signaling. To further explore the broader impact of BPA on neuronal function, we next investigated its effects on the neurite network. Several studies have demonstrated that chronic exposure to non‐cytotoxic doses of BPA impairs neuronal morphology across different models. Specifically, neurite length, branching, and arborization complexity are affected, with significant effects observed even at non‐cytotoxic micromolar concentrations, in both rodent and human neuronal systems [12, 42, 43, 44, 45]. In our cholinergic model, 24 h BPA exposure resulted in alterations of neurite number, length, and arborization as evidenced by morphometric analysis, confirming that also human cholinergic neurons can be affected by nontoxic dose of BPA with effects on neuritic complexity. Given the observed impairment in neuritic architecture, we next examined whether BPA exposure also impacts synaptic integrity in cholinergic neurons. In our cholinergic model, 24 h BPA exposure resulted in alterations to synapses, as evidenced by the expression levels of synaptic proteins SYT, SYP, and PSD‐95. A similar treatment of 24 h with a 100 μM concentration of BPA resulted in a decreased level of SYP and PSD‐95 in mouse cerebral cortex neurons [12]. Notably, in the same study, the structural remodeling of dendritic spines and the reduction in excitatory synapse number were directly linked to impaired synaptic transmission and cognitive deficits in vivo, supporting a causal relationship between morphological alterations and functional impairment [12]. The observed changes in synaptic protein expression and neuritic architecture indicate impaired synaptic activity, which may converge with mechanisms described in other contexts. In AD, in vivo SV2A PET imaging shows that synaptic density loss in cortical and hippocampal regions correlates with reduced functional and structural connectivity and with cognitive decline [46]. At the molecular level, the postsynaptic scaffolding protein PSD‐95 is a key organizer of excitatory synapses, controlling AMPA receptor incorporation during long‐term potentiation (LTP) and experience‐driven plasticity. Overexpression of PSD‐95 increases AMPAR‐mediated currents and mimics LTP, while dominant‐negative mutants block AMPAR delivery, demonstrating that structural assembly at the postsynaptic density is essential for functional potentiation [47]. PSD‐95 is also required for stabilizing potentiated synapses: its knockdown reduces AMPAR currents, prevents developmental increases in functional synapse number, and increases spine instability despite preserved LTP induction [48]. Although in the present study we did not aim to investigate the link between structural changes and functional impairment directly, the evidence above provides strong support for such a relationship, suggesting that structural synaptic alterations are highly likely to translate into functional abnormalities, even if our cellular model differs from those described. In this regard, although the underlying mechanisms may be different in our neuronal model, the combination of altered synaptic protein expression, reduced neuritic arborization, and cholinergic system dysregulation strongly suggests a close association with synaptic malfunction.

This provides a solid rationale for investigating the expression of activity‐dependent genes following high KCl‐induced depolarization in our model, as a means to assess potential alterations in synaptic plasticity mechanisms [29]. We demonstrated that short‐term treatment with BPA influenced the expression of all three analyzed genes after KCl‐induced depolarization. It is noteworthy that, already at the end of BPA treatment, there is a considerable difference in *NR4A1* gene expression, with a higher transcript level in BPA‐treated neurons compared to controls. This is likely explained by the fact that it is an estrogen‐inducible gene, as previously documented in myoblastic cells [49]. Indeed, Nr4a family proteins have been demonstrated to protect neurons from damaging stimuli and could serve as downstream effectors that contribute to the neuroprotective effects of CREB [50, 51]. Following KCl‐induced depolarization, the expression of this gene is disrupted as a consequence of alterations in synaptic activity. We also analyzed the expression of *LINC00473*, a primate‐specific long noncoding RNA (lncRNA) regulated by synaptic activity [52] and influenced by BDNF signaling [53]. Notably, this gene is downregulated in the prefrontal cortex of depressed females [54]. In our experiments, the increased expression of this gene after KCl stimulation is blunted in BPA‐treated neurons. The alteration of the expression level of this activity‐regulated gene under brief BPA treatment represents an interesting molecular link between BPA and depression, worthy of further investigation. A similar impairment in depolarization‐induced response was also observed for the expression of the BDNF gene. BDNF is essential for the formation and maintenance of activity‐dependent synaptic connections [55, 56, 57], and in general, it exerts neuroprotective effects. The reduction of BDNF expression in BPA‐treated neurons downstream of synaptic activity can have several significant consequences. It may lead to impairments in synaptic plasticity, affecting learning and memory processes. Decreased BDNF levels are also linked to mood disorders, cognitive decline, and, if persistent, to neurodegenerative diseases, as BDNF plays a crucial role in supporting neuron survival, growth, and the formation of new synapses.

Our findings are consistent with previous reports linking BPA exposure to adverse neurodevelopmental and neurodegenerative outcomes. In particular, the alterations in activity‐dependent gene expression that we observed are consistent with studies showing that prenatal exposure to BPA increases the risk of neuropsychiatric disorders in childhood, including depression, anxiety, and social and cognitive impairments commonly associated with autism spectrum disorder (ASD) [58, 59]. Furthermore, the reduced expression of BDNF and LINC00473 in our model is in line with evidence that BPA exposure contributes to neurodegeneration through mechanisms such as oxidative stress, neuroinflammation, and synaptic dysfunction [16, 17]. Importantly, our data extend these observations by demonstrating that even brief, non‐cytotoxic exposures interfere with the transcriptional programs that sustain neuronal plasticity and health. This suggests that the dysregulation of genes implicated in mood regulation and synaptic maintenance may represent an early molecular event bridging environmental BPA exposure to later brain dysfunction. Our data contribute to this understanding by showing that BPA interferes with activity‐regulated gene expression, an essential mechanism for synaptic plasticity and neuronal communication. Disruption of genes such as *NR4A1*, *LINC00473*, and *BDNF* following BPA treatment indicates that even brief exposures to non‐cytotoxic concentrations can impair the cellular programs that govern neuronal adaptation and health. Since these genes are implicated in both mood regulation and synaptic maintenance, their dysregulation may serve as an early molecular signature linking environmental exposure to later brain dysfunction. Overall, this supports the hypothesis that alterations in activity‐dependent gene expression represent an initial and potentially pivotal step in the cascade of events leading from environmental BPA exposure to impaired brain function, mood disorders, and possibly the neurodegenerative processes underlying diseases such as Alzheimer's.

## 5 Conclusion

The findings of this study offer novel insights into the impact of BPA exposure on synaptic function in a human neuronal model. In particular, brief exposure to BPA in a human cell model resulted in alterations to dendritic morphology, affecting synaptic protein expression and ultimately synaptic activity. These findings may contribute to our understanding of the potential role of BPA in various neuronal disorders. Our model can serve as an experimental platform to elucidate the molecular mechanisms underlying brain disorders, including depression, schizophrenia (SCZ), ASD, and epilepsy, among others. It can also be used to investigate neurodegenerative diseases that are preceded by alterations in synaptic activity, such as AD.

## Author Contributions

Anna Maria Carrese and Rossella Vitale performed most of the experiments. Manuela Turco performed some preliminary experiments. Natasha Petecca performed experiments reported in Figure 3. All authors performed data analysis. Anna Maria Carrese, Luisa Cigliano, Emilia Vitale, and Aldo Donizetti wrote the manuscript. Aldo Donizetti conceived the study. Luisa Cigliano, Francesco Aniello, and Aldo Donizetti were responsible for obtaining funding for the research project. All authors contributed to the finalization and approved the content of the manuscript.

## Conflicts of Interest

The authors declare no conflicts of interest.

## Supporting information

Figure_S1.

## Data Availability

The data that support the findings of this study are available from the corresponding authors upon reasonable request.

## References

[1] H. Inadera , “Neurological Effects of Bisphenol A and Its Analogues,” International Journal of Medical Sciences 12, no. 12 (October 2015): 926–936, 10.7150/ijms.13267. 26664253 PMC4661290

[2] A. Konieczna , A. Rutkowska , and D. Rachoń , “Health Risk of Exposure to Bisphenol A (BPA),” Roczniki Panstwowego Zakladu Higieny 66, no. 1 (2015): 5–11, PMID: 25813067. 25813067

[3] T. Geens , D. Aerts , C. Berthot , et al., “A Review of Dietary and Non‐Dietary Exposure to Bisphenol‐A,” Food and Chemical Toxicology 50, no. 10 (October 2012): 3725–3740, 10.1016/j.fct.2012.07.059. 22889897

[4] W. V. Welshons , S. C. Nagel , and F. S. vom Saal , “Large Effects From Small Exposures. III. Endocrine Mechanisms Mediating Effects of Bisphenol A at Levels of Human Exposure,” supplement, Endocrinology 147, no. S6 (June 2006): s56–s69, 10.1210/en.2005-1159. 16690810

[5] F. Acconcia , V. Pallottini , and M. Marino , “Molecular Mechanisms of Action of BPA,” Dose‐Response 13, no. 4 (October 2015): 1559325815610582, 10.1177/1559325815610582. 26740804 PMC4679188

[6] H. Batista‐Silva , K. Rodrigues , K. R. S. de Moura , et al., “In Vivo and In Vitro Short‐Term Bisphenol A Exposures Disrupt Testicular Energy Metabolism and Negatively Impact Spermatogenesis in Zebrafish,” Reproductive Toxicology 107 (January 2022): 10–21, 10.1016/j.reprotox.2021.11.001. 34775058

[7] X. Liu , H. Sakai , M. Nishigori , et al., “Receptor‐Binding Affinities of Bisphenol A and Its Next‐Generation Analogs for Human Nuclear Receptors,” Toxicology and Applied Pharmacology 377 (August 2019): 114610, 10.1016/j.taap.2019.114610. 31195007

[8] T. Qin , X. Zhang , T. Guo , et al., “Epigenetic Alteration Shaped by the Environmental Chemical Bisphenol A,” Frontiers in Genetics 11 (January 2021): 618966, 10.3389/fgene.2020.618966. 33505438 PMC7830874

[9] M. Nishikawa , H. Iwano , R. Yanagisawa , N. Koike , H. Inoue , and H. Yokota , “Placental Transfer of Conjugated Bisphenol A and Subsequent Reactivation in the Rat Fetus,” Environmental Health Perspectives 118, no. 9 (September 2010): 1196–1203, 10.1289/ehp.0901575. 20382578 PMC2944077

[10] Y. Sun , M. N. Nakashima , M. Takahashi , N. Kuroda , and K. Nakashima , “Determination of Bisphenol A in Rat Brain by Microdialysis and Column Switching High‐Performance Liquid Chromatography With Fluorescence Detection,” Biomedical Chromatography 16, no. 5 (August 2002): 319–326, 10.1002/bmc.161. 12210505

[11] J. D. Elsworth , J. D. Jentsch , C. A. Vandevoort , R. H. Roth , D. E. Redmond , and C. Leranth , “Prenatal Exposure to Bisphenol A Impacts Midbrain Dopamine Neurons and Hippocampal Spine Synapses in Non‐Human Primates,” Neurotoxicology 35 (March 2013): 113–120, 10.1016/j.neuro.2013.01.001. 23337607 PMC3660149

[12] S. A. Hyun , M. Y. Ko , S. Jang , et al., “Bisphenol‐A Impairs Synaptic Formation and Function by RGS4‐Mediated Regulation of BDNF Signaling in the Cerebral Cortex,” Disease Models & Mechanisms 15, no. 7 (July 2022): dmm049177, 10.1242/dmm.049177. 35781563 PMC9346518

[13] M. Yokosuka , R. Ohtani‐Kaneko , K. Yamashita , D. Muraoka , Y. Kuroda , and C. Watanabe , “Estrogen and Environmental Estrogenic Chemicals Exert Developmental Effects on Rat Hypothalamic Neurons and Glias,” Toxicology In Vitro 22, no. 1 (February 2008): 1–9, 10.1016/j.tiv.2007.07.003. 17761398

[14] Y. Ni , L. Hu , S. Yang , et al., “Bisphenol A Impairs Cognitive Function and 5‐HT Metabolism in Adult Male Mice by Modulating the Microbiota‐Gut‐Brain Axis,” Chemosphere 282 (November 2021): 130952, 10.1016/j.chemosphere.2021.130952. 34082316

[15] L. K. Pradhan , P. Sarangi , P. K. Sahoo , S. Kundu , N. R. Chauhan , and S. Kumar Das , “Bisphenol A‐Induced Neurobehavioral Transformation Is Associated With Augmented Monoamine Oxidase Activity and Neurodegeneration in Zebrafish Brain,” Environmental Toxicology and Pharmacology 97 (January 2023): 104027, 10.1016/j.etap.2022.104027. 36462733

[16] R. Rezg , S. El‐Fazaa , N. Gharbi , and B. Mornagui , “Bisphenol A and Human Chronic Diseases: Current Evidences, Possible Mechanisms, and Future Perspectives,” Environment International 64 (March 2014): 83–90, 10.1016/j.envint.2013.12.007. 24382480

[17] M. Takahashi , M. Komada , K. Miyazawa , S. Goto , and Y. Ikeda , “Bisphenol A Exposure Induces Increased Microglia and Microglial Related Factors in the Murine Embryonic Dorsal Telencephalon and Hypothalamus,” Toxicology Letters 284 (March 2018): 113–119, 10.1016/j.toxlet.2017.12.010. 29248573

[18] S. Suresh , A. Singh S , and C. Vellapandian , “Bisphenol A Exposure Links to Exacerbation of Memory and Cognitive Impairment: A Systematic Review of the Literature,” Neuroscience & Biobehavioral Reviews 143 (December 2022): 104939, 10.1016/j.neubiorev.2022.104939. 36328120

[19] T. Wang , C. Xie , P. Yu , et al., “Involvement of Insulin Signaling Disturbances in Bisphenol A‐Induced Alzheimer's Disease‐Like Neurotoxicity,” Scientific Reports 7, no. 1 (August 2017): 7497, 10.1038/s41598-017-07544-7. 28790390 PMC5548741

[20] C. Leranth , T. Hajszan , K. Szigeti‐Buck , J. Bober , and N. J. MacLusky , “Bisphenol A Prevents the Synaptogenic Response to Estradiol in Hippocampus and Prefrontal Cortex of Ovariectomized Nonhuman Primates,” Proceedings of the National Academy of Sciences of the United States of America 105, no. 37 (September 2008): 14187–14191, 10.1073/pnas.0806139105. 18768812 PMC2544599

[21] J. D. Elsworth , J. D. Jentsch , S. M. Groman , R. H. Roth , E. D. Redmond , and C. Leranth , “Low Circulating Levels of Bisphenol‐A Induce Cognitive Deficits and Loss of Asymmetric Spine Synapses in Dorsolateral Prefrontal Cortex and Hippocampus of Adult Male Monkeys,” Journal of Comparative Neurology 523, no. 8 (June 2015): 1248–1257, 10.1002/cne.23735. 25557059 PMC4390445

[22] C. Leranth , K. Szigeti‐Buck , N. J. Maclusky , and T. Hajszan , “Bisphenol A Prevents the Synaptogenic Response to Testosterone in the Brain of Adult Male Rats,” Endocrinology 149, no. 3 (March 2008): 988–994, 10.1210/en.2007-1053. 18048497 PMC2275360

[23] F. Hu , T. Li , H. Gong , et al., “Bisphenol A Impairs Synaptic Plasticity by Both Pre‐ and Postsynaptic Mechanisms,” Advanced Science (Weinheim, Baden‐Wurttemberg, Germany) 4, no. 8 (April 2017): 1600493, 10.1002/advs.201600493. 28852612 PMC5566242

[24] C. Y. Lee , S. A. Hyun , M. Y. Ko , et al., “Maternal Bisphenol A (BPA) Exposure Alters Cerebral Cortical Morphogenesis and Synaptic Function in Mice,” Cerebral Cortex 31, no. 12 (October 2021): 5598–5612, 10.1093/cercor/bhab183. 34171088

[25] X. Zhao and A. Bhattacharyya , “Human Models Are Needed for Studying Human Neurodevelopmental Disorders,” American Journal of Human Genetics 103, no. 6 (December 2018): 829–857, 10.1016/j.ajhg.2018.10.009. 30526865 PMC6288051

[26] L. Lopez‐Suarez , S. A. Awabdh , X. Coumoul , and C. Chauvet , “The SH‐SY5Y Human Neuroblastoma Cell Line, a Relevant In Vitro Cell Model for Investigating Neurotoxicology in Human: Focus on Organic Pollutants,” Neurotoxicology 92 (September 2022): 131–155, 10.1016/j.neuro.2022.07.008. 35914637

[27] C. Li , C. Sang , S. Zhang , S. Zhang , and H. Gao , “Effects of Bisphenol A and Bisphenol Analogs on the Nervous System,” Chinese Medical Journal 136, no. 3 (February 2023): 295–304, 10.1097/CM9.0000000000002170. 36848196 PMC10106255

[28] E. Meijering , M. Jacob , J. C. F. Sarria , P. Steiner , H. Hirling , and M. Unser , “Design and Validation of a Tool for Neurite Tracing and Analysis in Fluorescence Microscopy Images,” Cytometry, Part A 58, no. 2 (April 2004): 167–176, 10.1002/cyto.a.20022. 15057970

[29] A. M. Carrese , R. Vitale , M. Turco , et al., “Sustained Depolarization Induces Gene Expression Pattern Changes Related to Synaptic Plasticity in a Human Cholinergic Cellular Model,” Molecular Neurobiology 62, no. 1 (2025): 935–945, 10.1007/s12035-024-04262-w. 38941065 PMC11711863

[30] M. S. Spagnuolo , A. Donizetti , L. Iannotta , et al., “Brain‐Derived Neurotrophic Factor Modulates Cholesterol Homeostasis and Apolipoprotein E Synthesis in Human Cell Models of Astrocytes and Neurons,” Journal of Cellular Physiology 233, no. 9 (September 2018): 6925–6943, 10.1002/jcp.26480. 29323721 PMC13482536

[31] L. Cigliano , M. S. Spagnuolo , F. Boscaino , et al., “Dietary Supplementation With Fish Oil or Conjugated Linoleic Acid Relieves Depression Markers in Mice by Modulation of the Nrf2 Pathway,” Molecular Nutrition & Food Research 63, no. 21 (November 2019): e1900243, 10.1002/mnfr.201900243. 31398773

[32] H. E. Costa and E. Cairrao , “Effect of Bisphenol A on the Neurological System: A Review Update,” Archives of Toxicology 98, no. 1 (January 2024): 1–73, 10.1007/s00204-023-03614-0. 37855918 PMC10761478

[33] M. Sendra , M. Cavia‐Saiz , and P. Múñiz , “Are the BPA Analogues an Alternative to Classical BPA? Comparison Between 2D and Alternative 3D In Vitro Neuron Model to Assess Cytotoxic and Genotoxic Effects,” Toxicology 502 (February 2024): 153715, 10.1016/j.tox.2023.153715. 38211720

[34] Y. Lei , S. Wang , B. Ren , et al., “CHOP Favors Endoplasmic Reticulum Stress‐Induced Apoptosis in Hepatocellular Carcinoma Cells via Inhibition of Autophagy,” PLoS One 12, no. 8 (August 2017): e0183680, 10.1371/journal.pone.0183680. 28841673 PMC5571976

[35] D. Barrios , B. Bachhav , W. Carlos‐Alcalde , C. D. Llanos , W. Zhou , and L. Segatori , “Feedback‐Responsive Cell Factories for Dynamic Modulation of the Unfolded Protein Response,” Nature Communications 16, no. 1 (May 2025): 4106, 10.1038/s41467-025-58994-x. PMC12048557 40316547

[36] M. Šereš , L. Pavlíková , V. Boháčová , et al., “Overexpression of GRP78/BiP in P‐Glycoprotein‐Positive L1210 Cells Is Responsible for Altered Response of Cells to Tunicamycin as a Stressor of the Endoplasmic Reticulum,” Cells 9, no. 4 (April 2020): 890, 10.3390/cells9040890. 32268491 PMC7226765

[37] J. Nithianantharajah , H. Levis , and M. Murphy , “Environmental Enrichment Results in Cortical and Subcortical Changes in Levels of Synaptophysin and PSD‐95 Proteins,” Neurobiology of Learning and Memory 81, no. 3 (May 2004): 200–210, 10.1016/j.nlm.2004.02.002. 15082021

[38] T. Balbi , S. Franzellitti , R. Fabbri , M. Montagna , E. Fabbri , and L. Canesi , “Impact of Bisphenol A (BPA) on Early Embryo Development in the Marine Mussel *Mytilus galloprovincialis*: Effects on Gene Transcription,” Environmental Pollution 218 (November 2016): 996–1004, 10.1016/j.envpol.2016.08.050. 27569056

[39] R. P. Huang , Z. H. Liu , S. F. Yuan , H. Yin , Z. Dang , and P. X. Wu , “Worldwide Human Daily Intakes of Bisphenol A (BPA) Estimated From Global Urinary Concentration Data (2000‐2016) and Its Risk Analysis,” Environmental Pollution 230 (November 2017): 143–152, 10.1016/j.envpol.2017.06.026. 28649042

[40] K. Miyagawa , M. Narita , M. Narita , H. Akama , and T. Suzuki , “Memory Impairment Associated With a Dysfunction of the Hippocampal Cholinergic System Induced by Prenatal and Neonatal Exposures to Bisphenol‐A,” Neuroscience Letters 418, no. 3 (May 2007): 236–241, 10.1016/j.neulet.2007.01.088. 17467901

[41] P. Moyano , A. Flores , J. García , et al., “Bisphenol A Single and Repeated Treatment Increases HDAC2, Leading to Cholinergic Neurotransmission Dysfunction and SN56 Cholinergic Apoptotic Cell Death Through Ache Variants Overexpression and NGF/TrkA/P75NTR Signaling Disruption,” Food and Chemical Toxicology 157 (November 2021): 112614, 10.1016/j.fct.2021.112614. 34655688

[42] S. Gill and V. M. R. Kumara , “Comparative Neurodevelopment Effects of Bisphenol A and Bisphenol F on Rat Fetal Neural Stem Cell Models,” Cells 10, no. 4 (April 2021): 793, 10.3390/cells10040793. 33918242 PMC8103521

[43] X. Liang , N. Yin , S. Liang , et al., “Bisphenol A and Several Derivatives Exert Neural Toxicity in Human Neuron‐Like Cells by Decreasing Neurite Length,” Food and Chemical Toxicology 135 (January 2020): 111015, 10.1016/j.fct.2019.111015. 31812737

[44] H. Wang , L. Chang , J. S. Aguilar , S. Dong , and Y. Hong , “Bisphenol‐A Exposure Induced Neurotoxicity in Glutamatergic Neurons Derived From Human Embryonic Stem Cells,” Environment International 127 (June 2019): 324–332, 10.1016/j.envint.2019.01.059. 30953815

[45] X. Wu , A. Majumder , R. Webb , and S. L. Stice , “High Content Imaging Quantification of Multiple In Vitro Human Neurogenesis Events After Neurotoxin Exposure,” BMC Pharmacology and Toxicology 17, no. 1 (December 2016): 62, 10.1186/s40360-016-0107-4. 27903287 PMC5131404

[46] J. Zhang , J. Wang , X. Xu , et al., “In Vivo Synaptic Density Loss Correlates With Impaired Functional and Related Structural Connectivity in Alzheimer's Disease,” Journal of Cerebral Blood Flow & Metabolism 43, no. 6 (June 2023): 977–988, 10.1177/0271678X231153730. 36718002 PMC10196742

[47] I. Ehrlich and R. Malinow , “Postsynaptic Density 95 Controls AMPA Receptor Incorporation During Long‐Term Potentiation and Experience‐Driven Synaptic Plasticity,” Journal of Neuroscience 24, no. 4 (January 2004): 916–927, 10.1523/JNEUROSCI.4733-03.2004. 14749436 PMC6729816

[48] I. Ehrlich , M. Klein , S. Rumpel , and R. Malinow , “PSD‐95 Is Required for Activity‐Driven Synapse Stabilization,” Proceedings of the National Academy of Sciences of the United States of America 104, no. 10 (March 2007): 4176–4181, 10.1073/pnas.0609307104. 17360496 PMC1820728

[49] S. Nagai , K. Ikeda , K. Horie‐Inoue , S. Takeda , and S. Inoue , “Estrogen Signaling Increases Nuclear Receptor Subfamily 4 Group A Member 1 Expression and Energy Production in Skeletal Muscle Cells,” Endocrine Journal 65, no. 12 (December 2018): 1209–1218, 10.1507/endocrj.EJ17-0548. 30333364

[50] N. Volakakis , B. Kadkhodaei , E. Joodmardi , et al., “NR4A Orphan Nuclear Receptors as Mediators of CREB‐Dependent Neuroprotection,” Proceedings of the National Academy of Sciences of the United States of America 107, no. 27 (July 2010): 12317–12322, 10.1073/pnas.1007088107. 20566846 PMC2901488

[51] S. J. Zhang , M. Zou , L. Lu , et al., “Nuclear Calcium Signaling Controls Expression of a Large Gene Pool: Identification of a Gene Program for Acquired Neuroprotection Induced by Synaptic Activity,” PLoS Genetics 5, no. 8 (August 2009): e1000604, 10.1371/journal.pgen.1000604. 19680447 PMC2718706

[52] P. Pruunsild , C. P. Bengtson , and H. Bading , “Networks of Cultured iPSC‐Derived Neurons Reveal the Human Synaptic Activity‐Regulated Adaptive Gene Program,” Cell Reports 18, no. 1 (January 2017): 122–135, 10.1016/j.celrep.2016.12.018. 28052243 PMC5236011

[53] V. Aliperti and A. Donizetti , “Long Non‐Coding RNA in Neurons: New Players in Early Response to BDNF Stimulation,” Frontiers in Molecular Neuroscience 9 (March 2016): 15, 10.3389/fnmol.2016.00015. 26973456 PMC4773593

[54] O. Issler , Y. Y. van der Zee , A. Ramakrishnan , et al., “Sex‐Specific Role for the Long Non‐Coding RNA LINC00473 in Depression,” Neuron 106, no. 6 (June 2020): 912–926.e5, 10.1016/j.neuron.2020.03.023. 32304628 PMC7305959

[55] H. Jourdi , Y. T. Hsu , M. Zhou , Q. Qin , X. Bi , and M. Baudry , “Positive AMPA Receptor Modulation Rapidly Stimulates Bdnf Release and Increases Dendritic mRNA Translation,” Journal of Neuroscience 29, no. 27 (July 2009): 8688–8697, 10.1523/JNEUROSCI.6078-08.2009. 19587275 PMC2761758

[56] A. Holtmaat and K. Svoboda , “Experience‐Dependent Structural Synaptic Plasticity in the Mammalian Brain,” Nature Reviews Neuroscience 10, no. 9 (2009): 647–658, 10.1038/nrn2699. 19693029

[57] R. S. Duman , G. K. Aghajanian , G. Sanacora , and J. H. Krystal , “Synaptic Plasticity And Depression: New Insights From Stress and Rapid‐Acting Antidepressants,” Nature Medicine 22 3 (March 2016): 238–249, 10.1038/nm.4050. PMC5405628 26937618

[58] A. Miodovnik , S. M. Engel , C. Zhu , et al., “Endocrine Disruptors and Childhood Social Impairment,” Neurotoxicology 32, no. 2 (March 2011): 261–267, 10.1016/j.neuro.2010.12.009. 21182865 PMC3057338

[59] F. Perera , E. L. R. Nolte , Y. Wang , et al., “Bisphenol A Exposure and Symptoms of Anxiety and Depression Among Inner City Children at 10‐12 Years of Age,” Environmental Research 151 (November 2016): 195–202, 10.1016/j.envres.2016.07.028. 27497082 PMC5071142

