# Supplementary figures and images for: Bisphenol A Treatment Impairs Synaptic Function in Human Cholinergic Neurons
Source: J Biochem Mol Toxicol. 2025 Oct 6;39(10):e70558. doi: 10.1002/jbt.70558 (PMC12499904; doi:10.1002/jbt.70558)

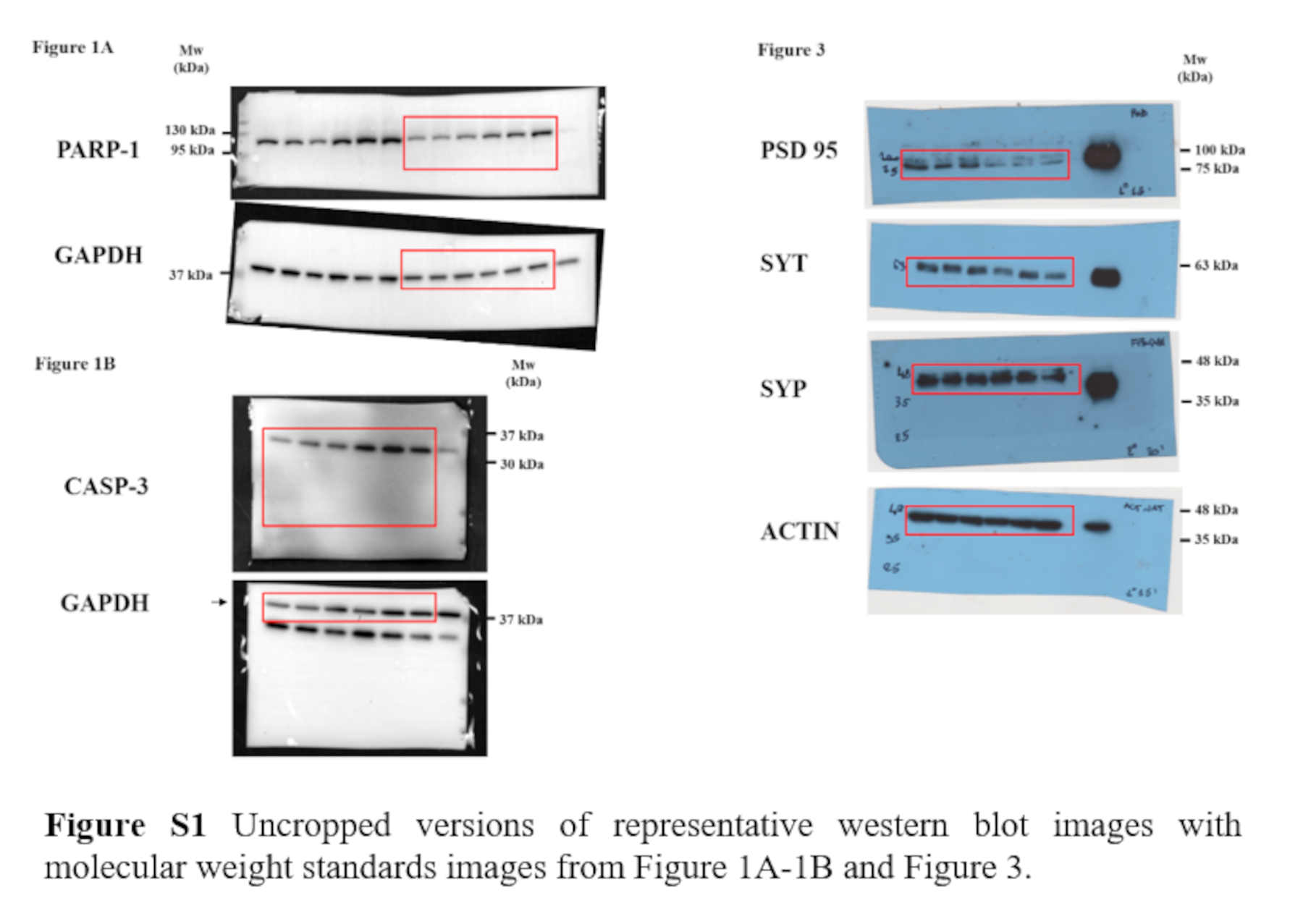

Supplement: Supplementary file 1 — Figure_S1. [file JBT-39-e70558-s001.tif]
